# Lingual nerve injuries: recognized complications or preventable errors?

**DOI:** 10.3389/froh.2025.1608292

**Published:** 2025-06-27

**Authors:** Frederic Van der Cruyssen, Robin Willaert, Michael Miloro

**Affiliations:** ^1^ Department of Oral and Maxillofacial Surgery, University Hospitals Leuven, Leuven, Belgium; ^2^ OMFS-IMPATH Research Group, KU Leuven, Leuven, Belgium; ^3^ Department of Oral and Maxillofacial Surgery, University of Illinois Chicago, Chicago, IL, United States

**Keywords:** lingual nerve injuries, complication, error, legal, perspective

## Abstract

Lingual nerve injuries (LNIs) represent a significant clinical challenge that can compromise speech, taste perception, and overall patient well-being. These may occur during third molar extractions, inferior alveolar nerve blocks, implant placement, root canal treatment and other dental, oral, and maxillofacial procedures. A major controversy centers on whether LNIs should be regarded as recognized complications—unavoidable events despite competent care—or potential negligence arising from substandard practice. Such classification hinges on factors including adherence to standard surgical protocols, informed consent, and timely postoperative assessment. Early detection using both qualitative and quantitative sensory evaluations allow prompt referral for microsurgical intervention, potentially improving outcomes if repairs are done within six months of injury. Conversely, lapses in diagnosis or management can lead to enduring disability, increased legal risk, and allegations of negligence. Evolving guidelines and advances in imaging and artificial intelligence may ultimately refine risk assessments, reduce complications, and alter standards of care. By recognizing the multifactorial nature of LNIs and adhering to rigorous surgical protocols, continuing education, and thorough documentation, healthcare professionals can optimize patient safety and potentially mitigate medico-legal challenges and enhance patient outcomes.

## Introduction

Lingual nerve injuries (LNIs) represent significant adverse events in oral and maxillofacial procedures, profoundly affecting patients' quality of life due to impaired sensation, speech, taste alterations, difficulty in mastication, and overall oral dysfunction (1). Patients experiencing LNIs often report substantial psychological distress further negatively impacting their social interactions and quality of life (2). Despite considerable advances in surgical techniques, diagnostic capabilities, and improved imaging technology, LNIs persist, highlighting the importance of understanding their preventability and medicolegal implications. Recently, the American Law Institute established a new legal standard, which now emphasizes “reasonable care” based on contemporary evidence rather than traditional customary practices (3). This perspective paper analyzes LNIs within the context of this updated medico-legal framework, incorporating anatomical, procedural, and technological factors to inform clinical practice, risk management, and legal accountability.

### Incidence and clinical relevance

LNIs might occur during routine procedures, including third molar extractions, inferior alveolar nerve blocks, placement of dental implants, periodontal surgeries, and endodontic treatments (4). The reported frequency of the two main causes of permanent lingual nerve damage varies but is often cited between 0.2% and 2.6% in lower third molar surgeries, and from 1:27,415 to 1:13,800,970 after inferior alveolar nerve blocks (5, 6). Temporary nerve injuries occur more frequently but still significantly impact patient's experience and healthcare consumption (7). Despite being relatively uncommon, the persistent nature of these injuries and associated morbidity necessitates stringent preventive and management strategies. Nerve injuries often result in medicolegal actions, further underscoring the importance for clinicians to adhere to evolving best practices and standards of care (8).

### Anatomical variability

A major challenge in preventing LNIs is the anatomical variability of the lingual nerve, particularly in its relationship to the mandibular third molars and the lingual cortex (9, 10). In certain cases, the nerve may lie in direct contact with or in close proximity to the lingual cortical bone or third molar, increasing the risk of injury during surgical extraction. The lingual nerve cannot be visualized intraoperatively during routine oral surgical procedures, as it lies in a different anatomical plane outside the surgical field. Consequently, the surgeon is unaware of its true location. Therefore, a surgical technique must be employed that minimizes the risk of nerve injury by accounting for the nerve's theoretical anatomical course.

The role of lingual flap retraction or protection during third molar surgery has also been extensively discussed in literature. Although intended to reduce LNI risk, its efficacy remains controversial, and others have shown it might instead increase the risk of temporary LNI (6). Several systematic reviews show conflicting results and suggest that these techniques do not consistently decrease injury incidence, necessitating surgeon-specific judgment based on case complexity (11–14).

Other surgical factors such as impaction level and angulation increase surgical difficulty and the likelihood of nerve injury (15). Similarly, improper presurgical planning in dental implant placement can elevate nerve injury risks. Patient traits including psychological factors, such as high anxiety and pain catastrophizing, may also influence patient outcomes and have been shown to be the most important factors in predicting chronicity after an injury has occurred (16–18).

Importantly, standard preoperative imaging modalities, including panoramic radiography and cone beam CT (CBCT), cannot visualize the lingual nerve due to its location solely within soft tissue planes, further complicating accurate preoperative risk assessments and necessitating heightened intraoperative vigilance.

Lingual cortical plate perforations, sometimes presented as indicators of negligence on post-injury CBCT images, may reflect natural anatomical variations rather than surgical trauma. Lingual plate defects or perforations occur naturally in 34%–65% of cases, related to the extreme thinness of the lingual cortical plate rather than surgical technique errors (19, 20).

Consequently, even with meticulous surgical planning and careful execution, the risk of nerve injury cannot be eliminated.

### Technological innovations and clinical applications

Technological advancements such as Magnetic Resonance Neurography (MRN) and artificial intelligence (AI)-based predictive modeling offer potential improvements in preoperative risk assessment (17, 21).

Recently, MR neurography has been introduced to selectively visualize peripheral nerves, offering improved delineation of peripheral nerves such as the lingual nerve (22, 23). However, this MRI technique is not readily available and is associated with MRI related costs, limiting its routine clinical application. In addition, dental-dedicated MRI machines are currently under investigation, but they have yet to be introduced into clinical practice (24).

AI-driven analytics could further refine patient-specific risk stratification and recommend personalized surgical approaches. Nevertheless, current limitations in technology accessibility, cost, and reliability restrict widespread clinical implementation (25). Continued interdisciplinary research is essential for overcoming these barriers and integrating advanced technologies into routine practice.

### Legal standards and the reasonable care model

Historically, assessments of surgical negligence have centered on adherence to customary medical practices. However, recent revisions by the American Law Institute (ALI) mark a significant shift toward a “reasonable care” standard (3). This framework prioritizes evidence-based medicine and contemporary clinical knowledge over tradition, urging practitioners to align their decisions with the latest scientific advancements rather than prevailing habits.

This evolving legal paradigm has direct implications for the evaluation of LNIs. Under the reasonable care model, clinicians are expected to stay current with the latest research, clinical guidelines, and technological advancements. Reasonable care is defined as the level of knowledge, skill, and diligence that would be considered competent among similarly qualified professionals under comparable circumstances. This standard allows juries to override customary practices if these are found to lag behind contemporary evidence-based norms.

In this context, good clinical practice must encompass three key elements: thorough preoperative assessment, planning and informed consent, precise surgical execution, and appropriate postoperative management. Prior to any intervention, clinicians should clearly document that informed consent was obtained, explicitly discussing the potential risk of nerve damage. While informed consent helps reduce medicolegal exposure by ensuring patients are aware of inherent procedural risks, it is not a preventive measure in itself. Equally critical is the correct execution of the surgical procedure, performed in accordance with up-to-date standards and individualized risk assessments. Should a nerve injury occur, clinicians must conduct a neurosensory examination and ensure timely referral to appropriate specialists. Thus, the reasonable care model calls for an integrated approach that balances legal responsibility with clinical excellence across the full continuum of patient care.

It is also essential to emphasize that the indication for third molar removal must be clearly validated in clinical documentation. From a medicolegal standpoint, even when lingual nerve injuries are deemed technically unpreventable due to anatomical variability and adherence to proper technique, litigation may still arise if the extraction was not justified. A recent example from France illustrates this: a third molar extraction led to a lingual nerve injury that was surgically unavoidable, yet the procedure was deemed litigious because it contravened the 2019 guidelines issued by the French National Authority for Health, which recommended against removal in that specific clinical scenario (26). As such, adhering to evidence-based and guideline-supported indications for third molar removal is crucial to mitigate the risk of litigation, particularly under the evolving legal standards of “reasonable care”.

In addition to the technical and legal considerations outlined above, the development of a well-structured care pathway for lingual nerve injuries is important. Early recognition and timely referral remain critical, as microsurgical repair is most effective within the first six months post-injury (27, 28). To support this, healthcare systems must ensure adequate access to specialized care while empowering first-line providers—including general dentists, oral surgeons, and maxillofacial specialists—to identify nerve injuries early and initiate prompt referral. While access barriers such as limited specialist availability and administrative delays can complicate this process, these challenges underscore rather than replace the clinician's responsibility to act decisively when lingual nerve injury is suspected (7, 8). Strengthening clinical training and establishing referral pathways are essential components of best practice under the reasonable care model, fostering timely intervention and improved patient outcomes.

Within this legal and clinical framework, a more nuanced understanding of LNIs is warranted—one that moves beyond binary classifications of outcomes as either acceptable complications or negligent errors. LNIs should be conceptualized along a continuum. This approach allows for a more refined evaluation that recognizes the spectrum of causality and responsibility:

• Recognized Complications: These are injuries that occur despite full adherence to contemporary, evidence-based standards. They typically result from unavoidable anatomical variations or inherent procedural risks and fall within the expected, though unfortunate, range of outcomes.
• Gray Area Cases: These involve partial deviations from optimal practice—such as insufficient documentation, ambiguous communication, or outdated techniques—that do not constitute gross negligence but may reflect suboptimal care. These cases often require detailed, context-specific assessments to determine appropriateness.
• Preventable Errors: At the far end of the spectrum are injuries resulting from clear departures from the standard of reasonable care. These include significant technical lapses, inadequate preoperative planning, or disregard for established preventive protocols.

This continuum not only mirrors the expectations of the reasonable care framework but also reinforces the dynamic nature of clinical responsibility. It emphasizes the importance of ongoing adaptation to evolving medical standards, in contrast to a static reliance on customary norms.

Considering this shift, proactive strategies are essential to minimize the occurrence of LNIs. Central to prevention is the advancement of medical education and continuous professional education. Furthermore, cultivating a safety culture grounded in human factors awareness can reduce avoidable errors. Interdisciplinary research should continue to drive innovation in areas such as high-resolution imaging, AI-assisted risk prediction, and minimally invasive techniques—all of which have the potential to reduce both clinical complications and medicolegal exposure.

## Conclusion

Classifying lingual nerve injuries (LNIs) solely as complications or errors fails to capture the complexity of their clinical and ethical dimensions. Our continuum framework provides a more nuanced and accurate approach for understanding and managing these events. LNIs are shaped by a multifactorial interplay of anatomical variability, procedural risks, human factors, and shifting legal expectations. To navigate this complexity, oral and maxillofacial surgeons, along with other dental professionals, must remain informed by the latest clinical evidence, integrate appropriate emerging technologies, and commit to ongoing professional development. Equally important is clear, patient-centered communication—particularly regarding risk disclosure, informed consent, and postoperative care. When nerve injuries do occur, timely, transparent, and ethically grounded responses are essential to maintain patient trust, enable effective intervention, and uphold the highest standards of professional accountability.

## Data Availability

The original contributions presented in the study are included in the article/Supplementary Material, further inquiries can be directed to the corresponding author.
